# Challenges and Future Perspectives in Photocatalysis: Conclusions from an Interdisciplinary Workshop

**DOI:** 10.1021/jacsau.4c00527

**Published:** 2024-08-08

**Authors:** Sebastian B. Beil, Sylvestre Bonnet, Carla Casadevall, Remko J. Detz, Fabian Eisenreich, Starla D. Glover, Christoph Kerzig, Line Næsborg, Sonja Pullen, Golo Storch, Ning Wei, Cathleen Zeymer

**Affiliations:** a Stratingh Institute for Chemistry, University of Groningen, 9747 AG Groningen, The Netherlands; b Max Planck Institute for Chemical Energy Conversion, Stiftstraße 34-36, 45470 Mulheim an der Ruhr, Germany; c Leiden Institute of Chemistry, Leiden University, Gorlaeus Laboratories, PO Box 9502, 2300 RA Leiden, The Netherlands; d Department of Physical and Inorganic Chemistry, University Rovira i Virgili (URV), C/Marcel.lí Domingo, 1, 43007 Tarragona, Spain; e Institute of Chemical Research of Catalonia (ICIQ), The Barcelona Institute of Science and Technology, Avinguda dels Països Catalans, 16, 43007 Tarragona, Spain; f Energy Transition Studies (ETS), Netherlands Organization for Applied Scientific Research (TNO), Radarweg 60, 1043 NT Amsterdam, The Netherlands; g Department of Chemical Engineering and Chemistry & Institute for Complex Molecular Systems, Eindhoven University of Technology, 5600 MB Eindhoven, The Netherlands; h Department of Chemistry, Ångström Laboratory, Uppsala University, Box 523, 75120 Uppsala, Sweden; i Department of Chemistry, Johannes Gutenberg University Mainz, Duesbergweg 10-14, 55128 Mainz, Germany; j Department of Organic Chemistry, University of Münster, Correnstr. 40, 48149 Münster, Germany; k Homogeneous and Supramolecular Catalysis, Van ’t Hoff Institute for Molecular Sciences, University of Amsterdam, 1098 XH Amsterdam, The Netherlands; l Technical University of Munich (TUM), Lichtenbergstr. 4, 85747 Garching, Germany; m Center for Functional Protein Assemblies & Department of Bioscience, TUM School of Natural Sciences, Technical University of Munich, 85748 Garching, Germany

**Keywords:** Photocatalysis, abundant metal catalysts, photobiocatalysis, artificial photosynthesis, mechanistic studies, photoreactor homogeneity, eco-friendly processes, photochemistry for sustainability

## Abstract

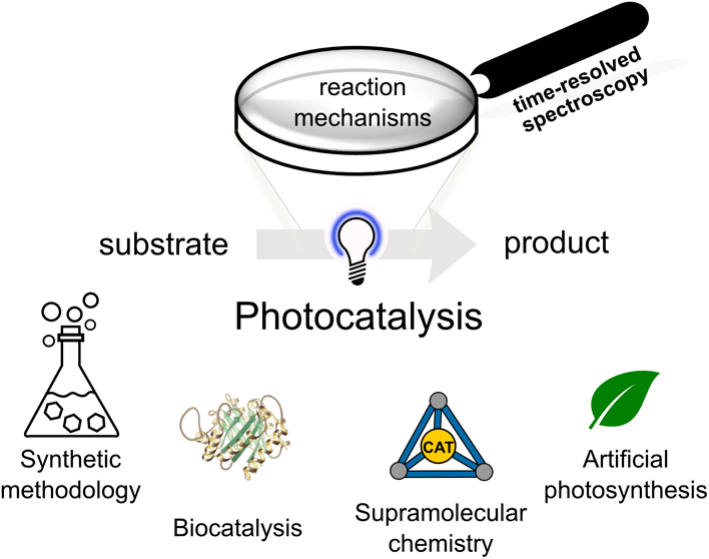

Photocatalysis is a versatile and rapidly developing field with applications spanning artificial photosynthesis, photo-biocatalysis, photoredox catalysis in solution or supramolecular structures, utilization of abundant metals and organocatalysts, sustainable synthesis, and plastic degradation. In this Perspective, we summarize conclusions from an interdisciplinary workshop of young principal investigators held at the Lorentz Center in Leiden in March 2023. We explore how diverse fields within photocatalysis can benefit from one another. We delve into the intricate interplay between these subdisciplines, by highlighting the unique challenges and opportunities presented by each field and how a multidisciplinary approach can drive innovation and lead to sustainable solutions for the future. Advanced collaboration and knowledge exchange across these domains can further enhance the potential of photocatalysis. Artificial photosynthesis has become a promising technology for solar fuel generation, for instance, via water splitting or CO_2_ reduction, while photocatalysis has revolutionized the way we think about assembling molecular building blocks. Merging such powerful disciplines may give rise to efficient and sustainable protocols across different technologies. While photocatalysis has matured and can be applied in industrial processes, a deeper understanding of complex mechanisms is of great importance to improve reaction quantum yields and to sustain continuous development. Photocatalysis is in the perfect position to play an important role in the synthesis, deconstruction, and reuse of molecules and materials impacting a sustainable future. To exploit the full potential of photocatalysis, a fundamental understanding of underlying processes within different subfields is necessary to close the cycle of use and reuse most efficiently. Following the initial interactions at the Lorentz Center Workshop in 2023, we aim to stimulate discussions and interdisciplinary approaches to tackle these challenges in diverse future teams.

## Introduction

In view of the intensified climate crisis and increasing resource scarcity, our society is in urgent need for new strategies to generate fuels, chemicals, and materials from renewable feedstocks. For future generations, action needs to be taken to develop more efficient transformations solely relying on renewable energy and without the emission of hazardous substances, such as harmful dye substances, heavy metals, and pharmaceutical residues, in natural (aquatic) ecosystems. Besides developing sustainable processes to build new molecules, the deconstruction, safe removal, and repurposing of building blocks and materials is thus of utmost importance for a sustainable future.

Currently, electrocatalysis appears to be a promising solution since renewable electricity is largely available through wind, water, and solar power.^1^ We believe that in a long-term vision, photocatalysis as a direct sunlight-driven process has the potential to contribute to a circular economy that combines both synthesis and chemical recycling of various chemicals, materials, and fuels.^2^ Photocatalysis also allows for the development of novel reaction routes via excited-state reactivity that are inaccessible through legacy (thermal and electrochemical) catalytic schemes based on ground-state pathways. Furthermore, excited states generated by light enable thermodynamically uphill reactions, which forms the basis for solar energy storage into fuels.^3−8^ Notably, the application of light-mediated chemical reactions has enormously increased in recent years, but the research field is by no means young. At the beginning of the 20th century, the pioneer Giacomo Ciamician already described the use of sunlight to drive chemical reactions–a vision that is still very timely more than a century later.^9^

The field of photochemistry has tremendously benefited from improved light sources, which are widely available today for experimental studies on a laboratory scale. With the relatively narrow-banded emission profiles of modern LEDs, photochemical reactions can be precisely controlled and studied, and can potentially be performed with a high level of reproducibility, if standardized protocols and equipment are used.^10−13^ Alternatively, solar simulators that mimic the solar spectrum enable the study of photocatalysts (PCs) relevant for solar fuel generation under realistic or real-world conditions on laboratory scale.

It is largely a consequence of this easy access to laboratory equipment that photochemical methods have become powerful methods in modern synthetic organic chemistry impacting the life science industry.^14^ In addition chemical recycling of polymers^15,16^ or degradation of waste products into environmentally harmless products,^17−19^ as well as solar fuel generation,^20,21^ greatly benefitted from these developments. The latter ultimately aims at using sunlight rather than artificial light setups.

**Figure 1:**
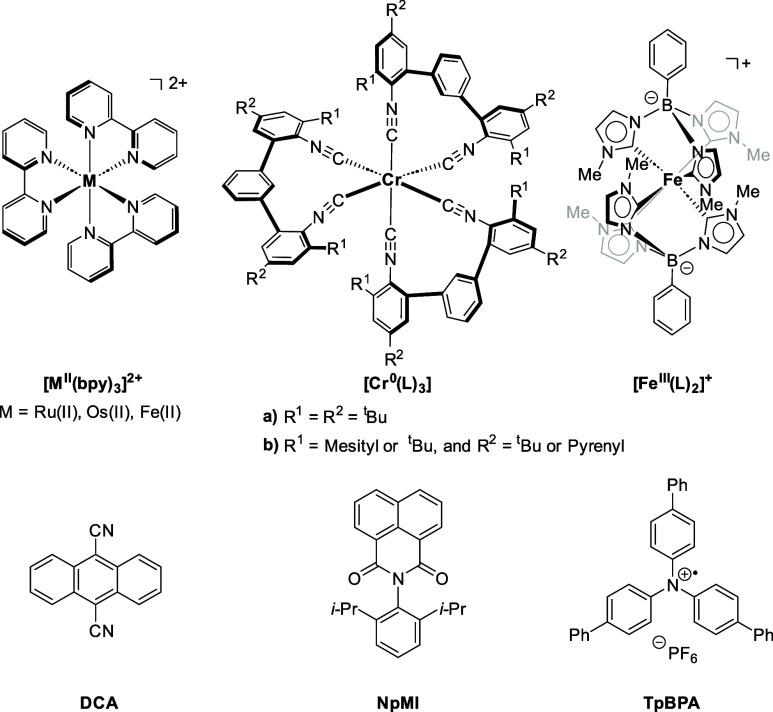
Examples of metal-based and organic photocatalysts. Top: Traditional polypyridyl complexes and novel first row transition metal catalysts have similar properties. Bottom: Organic photocatalysts can reach strong reducing (DCA and NpMI) or oxidizing (TpBPA) power.

Classical examples of photocatalysts, that are used across the whole field, traditionally include ruthenium- and iridium-based complexes,^22−24^ while more recently improvements have been achieved with purely organic catalysts that can reach high reductive and/or oxidative power (Figure 1).^25^ Nonetheless, new organic photocatalysts are required and should be designed with the aim to replace transition metal variants to add organocatalytic activity and take inspiration from Nature’s chromophores.^26−30^ Tunability of excited state properties is still a major challenge in the use of organic sensitizers and comes with additional synthetic constraints.^31^ However, metal complexes based on first-row and abundant transition metals are in many cases not competitive with the larger homologues (e.g., Ru, Os, or Ir) with respect to their photophysical properties and/or photostability.^32−36^ A recent exception is represented by emissive Cr(0) complexes [Cr^0^(L)_3_] with chelating isocyanides developed in the Wenger lab,^37^ which have properties similar to the red-light absorbing benchmark complex [Os(bpy)_3_]^2+^. While the isocyanide approach to stabilize long-lived excited states is clearly elegant, ligand and complex synthesis are rather challenging and necessitate experienced synthetic chemists.^38^ For the emerging class of Earth-abundant photoactive metal complexes, more complex and sophisticated ligands are frequently required, and they suffer from time-consuming synthesis,^39^ where heavy-metal photocatalysts utilize simplified and commercial ligands. It is worth mentioning that the tridentate carbene ligand of the versatile [Fe^III^(L)_2_]^+^ sensitizer reported by Wärnmark and coauthors in 2019 can be prepared in a single synthetic step.^40^ The resulting complex with a sufficiently long nanosecond lifetime of the excited charge-transfer state found already some promising applications in photoredox chemistry and photocatalysis.^36^

Modern approaches in the field of synthetic methodology are diverse, and new methods are constantly emerging.^41−45^ One particular focus was on achieving extremely strong reductive or oxidative power locally by starting with open-shell photocatalysts, which culminated in the identification of photochemical methods powerful enough to reduce^46,47^ or oxidize unsubstituted benzene.^48^ Another aspect is stereoselectivity, which has been recently achieved in reactions driven by both photoredox catalysis and triplet energy transfer catalysis.^49−52^ Stereo- and regioselectivity can be achieved by a tunable outer coordination sphere that is provided by chiral synthetic ligands as well as protein scaffolds of enzymes or synthetic analogues, making use of noncovalent interactions.^51,53^ Thus, to combine photochemistry with enzymes or synthetic (bioinspired) systems is a very promising strategy to achieve selectivity control.^54,55^ Photocatalysis can also be combined with other catalytic functions that operate in the dark, allowing the setup of cascade reactions, leading to accessibility of more complex synthetic routes in one pot.^56,57^

In addition to synthetic applications, photocatalysis has also experienced increasing interest for use in deconstruction of plastic waste and other pollutants.^58,59^ When photocatalysts are exposed to (sun)light, they create highly reactive species that can effectively convert pollutants into harmless or reusable byproducts.^60^ Heterogeneous metal oxide photocatalysts, such as TiO_2_^61^ or ZnO,^62^ are effective catalysts for targeted photodegradation, but they require short wavelength irradiation since they only absorb in the UV region. While the degradation of some of the most persistent pollutants has been demonstrated on laboratory scale, real life (waste)water treatment requires further optimization in terms of reactor design, rationalizing catalyst reactivity, and catalyst immobilization for recycling.^63^

Herein, we discuss the increasingly broad field of photocatalysis from different perspectives. We believe that a combination of various disciplines is required to address each of the above-mentioned challenges, with disciplines including traditional synthetic methodology development, spectroscopic investigations, and novel concepts derived from biology and bioinspired systems. Bridging various fields of photocatalysis will create synergy and thus advance the development of an overall more sustainable and circular production system. Furthermore, some techno-economic insights are provided to assess the potential of photocatalysis as a “green alternative”, which is a common praise of photocatalysis in the literature. The underlying discussions were kicked off during a workshop organized by the lead authors at the Lorentz Center in Leiden, the Netherlands, in 2023.

## Importance of Mechanistic Understanding

**Figure 2:**
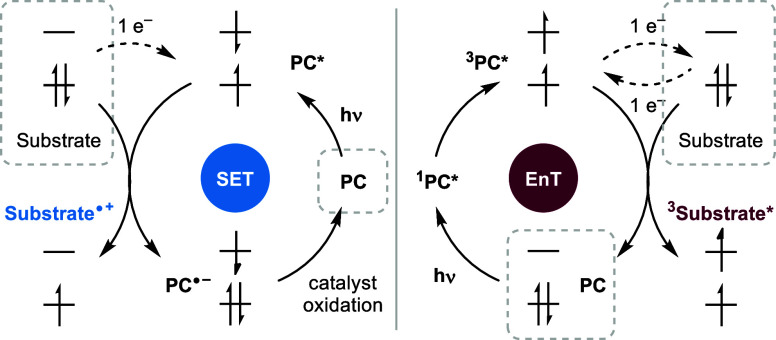
Schematic overview of reductive quenching via single-electron transfer (SET) (oxidative quenching resulting in a **substrate**^**•–**^ not shown) and energy transfer (EnT) mechanisms from the respective ground states (dashed lines) and photocatalysts (PC).

For the efficient use of photocatalysis and rational design of novel synthetic routes, a detailed fundamental understanding of the underlying mechanism is of utmost importance. Fundamentally, photochemistry can be divided into (single) electron transfer (SET) or photoredox transformations and energy transfer (EnT) or sensitization processes, depending on whether there is a net electron transfer between the excited photocatalyst and the substrate (Figure 2). Both reductive and oxidative quenching of photocatalysts benefit from significantly altered redox potentials. Energy transfer from photocatalysts in their excited triplet states typically occurs via the *Dexter* mechanism (a two-electron exchange process), which *inter alia* converts a substrate from its ground state singlet to the excited triplet state.^64^ To probe this, phosphorescence spectra are used to obtain experimental values for the triplet energy of substrates and catalysts, which allows the assessment of whether a particular substrate/catalyst combination is suitable. It is an intrinsic challenge that catalysts with high triplet energies typically require short wavelength irradiation, which may lead to direct substrate activation and thus unwanted side reactions. Desirable photosensitizers also have high intersystem crossing quantum yields, which implies that their excited triplet state is efficiently populated after initial excitation.^64,65^ Better design criteria are important to expand the applications of visible-light photosensitizers (PSs).^66^

**Figure 3:**
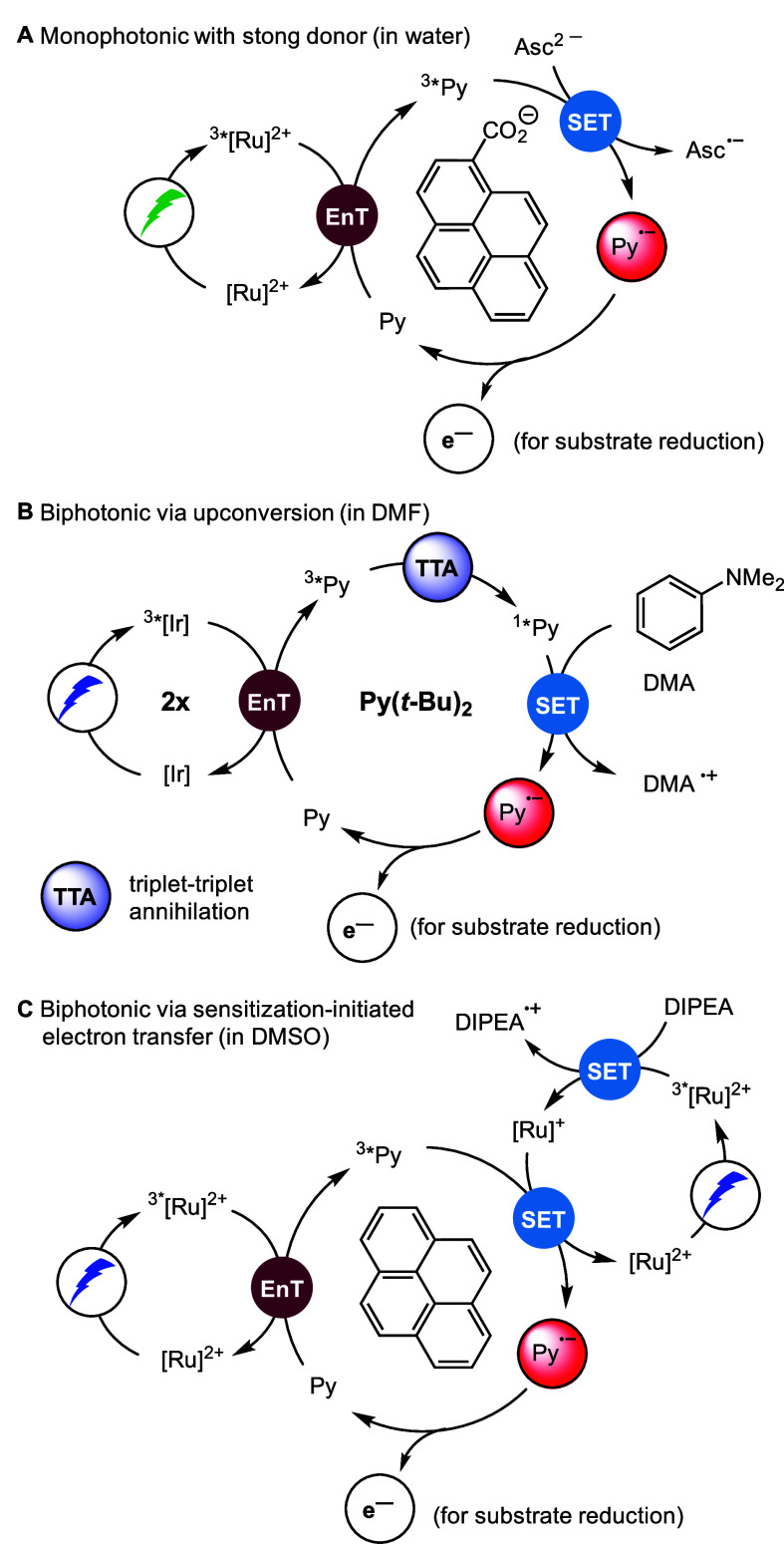
Photocatalytic systems (A, B, C) capable of producing a pyrene radical anion Py^•–^. Clear mechanistic evidence was obtained for these systems by TA spectroscopy. Asc^2–^: ascorbate; DIPEA: diisopropyl ethylamine; [Ru]: [Ru(bpy)_3_]X_2_; [Ir]: Ir(ppy)_3_; Py(*t*-Bu)_2_: 2,7-di-*tert*-butylpyrene.

Intense research on photocatalytic mechanisms in the photocatalysis field over the past 15 years has revealed that the mechanistic diversity is often far more complex than the simplified direct one-electron substrate activation picture. An impressive example for that is given by photoreductions carried out with a three-component model system containing (i) a photoactive metal complex for harvesting visible light, (ii) a pyrene (Py) derivative as electron or energy acceptor, and (iii) a sacrificial electron donor. Depending on the solvent and the donor, clear evidence for at least three completely different mechanisms have been obtained (Figure 3).^67−70^ Importantly, this is not an exclusive example, as similar mechanistic diversity has been observed and controversially discussed for many more photocatalyst combinations. A full mechanistic understanding thus lays the foundation for the rational design of photocatalytic systems and the straightforward optimization of reaction parameters.^23,71−73^

The generated pyrene radical (Py^•–^) is a highly reducing species (−2.1 V vs SCE) being able to activate hard to reduce and therefore challenging substrates via single electron transfer. For its generation with visible light, an aqueous system with the ascorbate dianion as a very strong donor that is only present in alkaline solution (Figure 3A) can be used. Alternatively, with weaker and more conventional amine-based donors in organic solvents the accumulation of the energy of two visible photons is required for thermodynamic reasons.

Two inherently different two-photon mechanisms can finally lead to the key species Py^•–^: upconversion via sensitized triplet–triplet annihilation (Figure 3B) with reduction of the resulting singlet-excited pyrene and sensitization-initiated electron transfer in which the reduced Ru complex reacts with triplet-excited Py to yield Py^•–^. A synthetically useful approach relying on mechanism B in aqueous micelles (without oxygen removal) has been reported recently.^74^ Moreover, a system based on mechanism C with a pyrene covalently attached to a chiral phosphoric acid revealed that two-photon chemistry can be combined with asymmetric photoredox catalysis (Figure 3C).^75^ These impressive recent examples came out almost exactly 10 years after König’s pioneering paper on the conPET mechanism that initiated the field of multiphoton photocatalysis.^76^ We believe that both blue and red light driven two-photon (or multiphoton) strategies are most promising for broad applications in the near future. First, efficient high-power blue LEDs or diode lasers are widely available, and the highest excited-state energies and redox potentials can be reached when pooling photons from the high-energy edge of the visible spectrum (while still avoiding harmful UV light with its selectivity issues). Second, red photons are widely used for upscaling, as a result of the deep penetration depth of these low-energy photons. Selective activation with red light will lead to higher selectivity in photocatalyzed organic synthesis due to less interactions with other components in the reaction mixture and thus less side reactions.^77,78^ In addition, red light can penetrate deeper into biological tissues, which opens new possibilities for biological applications.^79,80^ Therefore, the development of red-light activated photocatalysts will also likely advance the field of bio-orthogonal or semiartificial photocatalysis.

In photocatalytic mechanisms, readily available emission-based techniques are frequently employed for quenching studies of the initially formed excited state. However, these techniques suffer from several limitations. First, efficient photocatalyst quenching does not necessarily indicate high reaction quantum yields. Chemically unproductive electron transfer quenching caused by so-called *in-cage recombination* (usually on a sub-nanosecond time scale) is among the most prominent reasons for this discrepancy.^81^ In this process, the geminate radical pair recombines unproductively before it can separate into reactive species for desired onward reactions. In several examples for which cage escape yields were reported, less than 10% of all photoredox quenching events lead to these (desired) reactive species.^81^ Once separated, diffusion-based recombination (usually on a microsecond time scale) can be avoided by irreversible bond cleavage as observed for reductive dehalogenations or, e.g., protonation/deprotonation events, thereby ensuring productive follow-up chemistry. Second, usually only singlet-excited states of organic chromophores and phosphorescent metal complex photosensitizers can be analyzed using emission spectroscopy. Transient absorption (TA) spectroscopy, either via laser flash photolysis or using a pump–probe setup, can be regarded as a more versatile technique capable of providing detailed mechanistic information.^82^ Not only does TA spectroscopy provide direct temporal quantification of photocatalytic intermediates, such as organic triplets and substrate- or catalyst-derived species in different redox and protonation states, and products, but direct observation of the reaction intermediates can provide the means to clearly delineate energy and electron transfer pathways. Additionally, TA spectroscopy is an excellent tool for studying the dual singlet and triplet mechanisms of organic photocatalysts, like acridinium-based ones.^83,84^ Understanding both the triplet and singlet reaction channels is crucial. With this in mind, concentration dependent outcomes of photocatalytic reactions can be rationalized as singlet quenching competes with catalyst triplet formation. We believe that such studies are most relevant for organic thermally activated delayed fluorescence (TADF) compounds, an emerging class of photocatalysts with inherent dual reactivity.^30^ Importantly, to gain realistic mechanistic insights, spectroscopic studies should be carried out under synthetic conditions, i.e., similar to those of the actual photocatalytic reactions.^69,70^

As TA spectroscopy often requires expensive equipment and extensive training, it is not always available to all chemists interested in photocatalysis.^85^ However, we urge the reader to seek for collaborations. In addition to TA spectroscopy, irradiation experiments are a powerful tool in providing key mechanistic information for a photocatalytic system yet require less complex experimental equipment. We recommend following the sensitivity assessment for photoreactions initially suggested by Glorius.^86^ For instance, the assessment contains light power-dependent product yield studies, which can reveal whether a single photon or the consecutive absorption of at least two photons is required per catalytic turnover.^87^ Generally, reporting the number of absorbed photons obtained by actinometry and respective reaction quantum yield should be standardized in the field.^88−90^ Furthermore, reaction kinetics by irradiation studies, like time-dependent substrate conversion or intermediate or product formation, have the potential (i) to identify key reaction intermediates or even catalytically active species and (ii) to reveal that the initial photocatalyst may not be the direct origin of the catalytically active species.^91^ In the latter case, the observation of a lag phase indicates that light driven preactivation (or even photo-decomposition) of the catalyst takes place.^92^ Insightful mechanistic and kinetic studies during irradiation can be carried out using standard analytical equipment e.g., mass spectrometry^93,94^ and NMR.^95,96^ For example, combined irradiation-NMR experiments are available to an increasing number of chemists. This technique can be used to quantify the quantum yield and kinetics on the minutes to hours time scale to infer an overall mechanistic picture.^97^ Nevertheless, TA spectroscopic techniques will still be needed to detect short-lived intermediates, which is necessary to elucidate discrete mechanistic reaction steps. The knowledge gained from irradiation and faster TA mechanistic studies is needed to guide the rational design. In our view, this synergy will trigger the development of novel and potentially more efficient photocatalytic systems.

## Photocatalysts with Extreme Redox Potentials

Many potentially interesting substrates, such as aryl and alkyl halides, require high reduction potentials in order to be activated. It has been demonstrated that radical anions of common organic photosensitizers can achieve reduction potentials comparable to alkali metals under irradiation.^98,99^ In a similar fashion, radical cation photocatalysts can achieve strongly oxidizing excited states.^100^

**Scheme 1:**
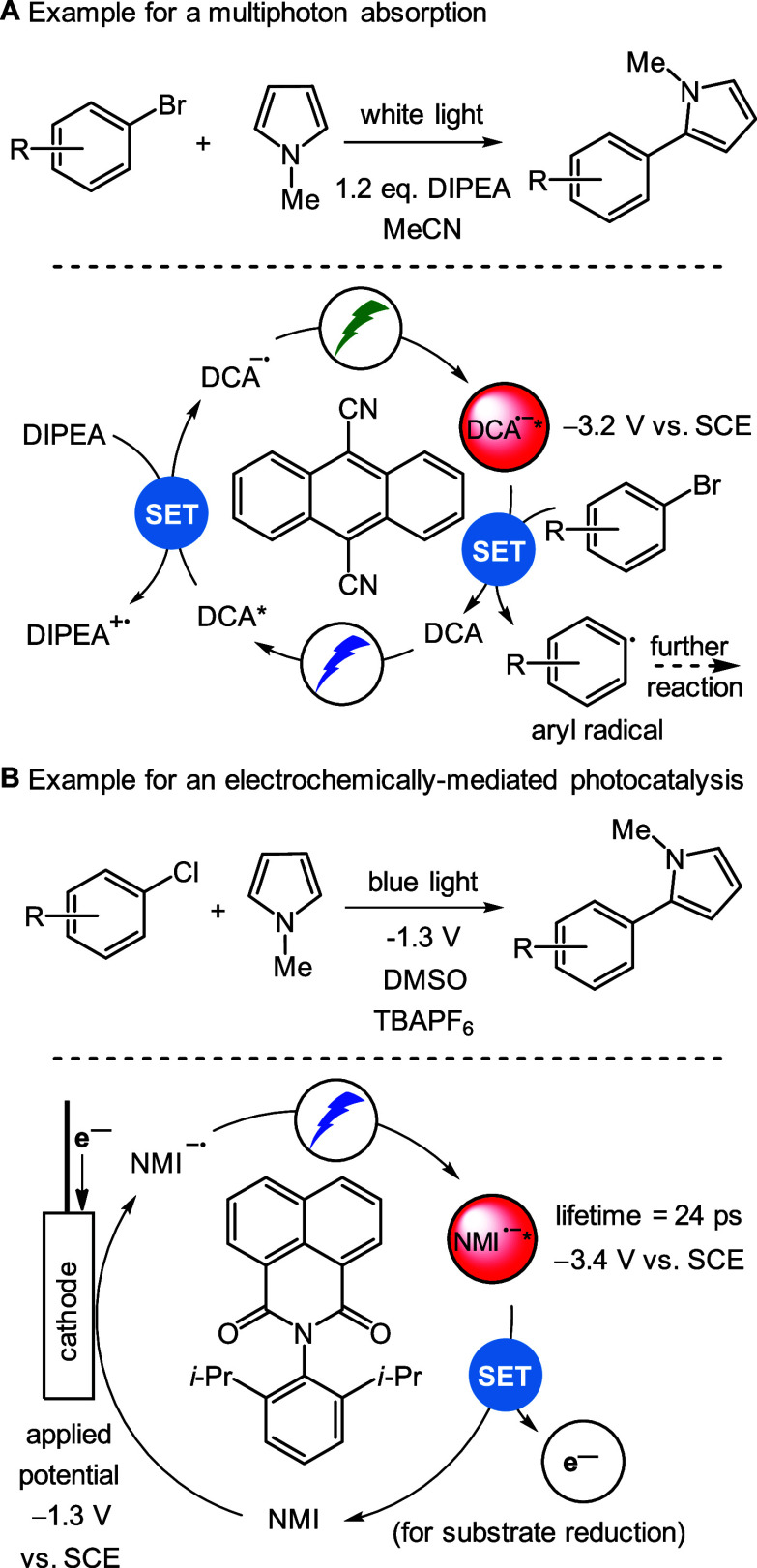
Examples of (a) Multiphoton Absorption Mechanism with DCA and (b) Electrochemically Mediated Photocatalysis with NMI

A convenient method to form such radical anions from the neutral precursors is electrochemically mediated photocatalysis. Here, radical anions of the photocatalysts are generated at an electrode prior to excitation. Excited state radical anions achieve excited state reduction potentials significantly stronger than those of their neutral analogues. As an example, the 9,10-dicyanoanthracene anion (DCA^–•^) reaches an excited state reduction potential of −3.2 V vs SCE.^98^ In a seminal study by Wangelin and Pérez-Ruiz, DCA^–•^ was generated via dichromatic absorption:^101^ Upon initial excitation with blue light and reductive quenching with DIPEA, the DCA^–•^ radical anion was generated photochemically, which was then consecutively excited by green light to achieve the strongly reducing excited intermediate *DCA^–•^, that could activate aryl bromides and cleave the C(sp^2^)–Br bond (Scheme 1A). Later on, Lambert and Lin showed that DCA^–•^ can also be generated electrochemically, and then follow a similar catalytic cycle upon excitation.^98^ The generated aryl radicals can either form the dehalogenation products via hydrogen atom abstraction (HAT) from the solvent or be used in coupling reactions as shown in Scheme 1. Mechanistic studies on photocatalytic schemes employing open-shell radical photocatalysts have revealed that excited states of such radical photocatalysts typically feature picosecond lifetimes, which in principle precludes diffusion-controlled reactions. To rationalize the observed reactivity, many studies suggest the formation of a radical anion–substrate complex.^102^ Very recently, spectroscopic evidence on the formation of such a dicyanoarene anion radical complex has been presented by Wenger:^103^ using transient absorption spectroscopy (TAS) quenching studies with the ultrashort DCA^–•^, they found evidence for the existence of such a complex. Analogously, radical cation photocatalyst–substrate preassociation has been suggested and experimentally proven with TAS^104^ and steady state ESR/UV studies^100^ by Hauer and Barham for the cationic TpBPA^+^ photocatalyst.

Polyaromatic fused imides such as perylene-diimide (PDI) and naphthalene monoimide (NMI) feature interesting photocatalytic properties in both their neutral and anionic states.^105,106^ While PDI has been mostly used in the context of multiphoton absorption, NMI has been employed in electrochemically mediated photocatalysis (Scheme 1B). While their reactivity in several reactions such as dehalogenation and C–C cross coupling has been illustrated in various reports,^76,107,108^ the exact mechanisms are still unclear and under intense debate.^73,92,109,110^ For example, for PDI an initially proposed dual blue photon absorption seems unlikely because the PDI^–•^ radical anion generated upon excitation with blue light followed by reductive quenching does not absorb in the blue region. Some studies suggest that decomposition products of PDI are the truly active catalyst, although the nature and properties of these remain unclear. A special case is the NMI photocatalyst. Initial reports by Wickens^107^ and Barham^102^ on the reactivity of the excited radical anion NMI^–•^ were questioned by Nocera, due to its ultrashort excited state lifetime. Nocera argued that the doubly reduced and protonated Meisenheimer complex NMI(H)^−^ may be a more realistic catalyst mostly due to its significantly longer lifetime enabling diffusion controlled processes.^111^ It should be noted that NMI(H)^−^ in this report was generated by NaBH_4_ as a chemical reductant and spectro-electrochemistry at higher potentials than what was used in the report by Wickens, which makes a fair comparison difficult.

Lastly, the group of Lambert found that cyclopropenium ions are versatile catalysts with extreme redox potentials in numerous organic transformations and can also be activated by a combined use of electrochemical activation followed by visible light excitation.^112^ Their developments culminated in the remarkable di- and trioxygenation of saturated small molecules.^113^

It is expected that further developments and understanding of the interplay of electrochemical oxidation or reduction followed by light excitation of the respective radical cations and anions will facilitate future advancements of photoelectrochemical transformations.^69,73^

## Reproducibility in Photocatalytic Reactions

On a practical note, optimizing conditions for photocatalytic processes (light intensity, wavelength, reaction temperature, reactor type, etc.), as well as comparing or reproducing different photocatalytic procedures between different laboratories, is a significant obstacle. The challenge mostly arises from the lack of homogeneity in reactor design and light irradiation setups between different research groups as well as a lack of a generally accepted roadmap to report measurable parameters. Besides the catalytic turnover number (TON) and the product yield of a reaction that are normally reported, reporting of additional measurable parameters is needed that can help to normalize and standardize photocatalytic procedures. As such, reporting the number of photons that arrive at the reaction and the corresponding quantum yield to quantify the efficiency of a photocatalytic reaction will facilitate direct comparison of photocatalytic protocols and results.^114,115^ The number of absorbed photons can be quantified by standard actinometry protocols.^88−90^ Additionally, the detailed description of the setups (light source, distance from the reactions, etc.) is particularly important to facilitate the reproducibility of the photocatalytic procedures independent of location.^3,116,117^ This is crucial for several reasons: (i) to gain insights into the reactivity induced by light; (ii) for conducting detailed mechanistic investigations; and (iii) to establish reliable and reproducible protocols across diverse laboratories. Moreover, this uniform light exposure plays a key role in advancing and refining laboratory protocols with the goal of facilitating their transition toward future industrial-scale applications.^118−121^

An additional challenge leading to irreproducibility is that many photocatalytic reactions are heterogeneous in nature. Either insoluble inorganic bases are used in an organic solvent (e.g., Cs_2_CO_3_ in DMF)^122^ or the photocatalysts themselves are poorly soluble (e.g., mesoporous graphitic carbon nitride)^123^ leading to inconsistencies due to precipitation or clogging. Whereas the latter simplifies recovery and reuse of the PC and the former avoids often toxic organic bases, both resemble heterogeneous particles of unknown size distribution, which affects light scattering and therefore causes lower consistency in reproducibility. The advances in heterogeneous photocatalysis have been reviewed and are outside of the scope of this Perspective.^124,125^ Despite the chemical necessity, a fully homogeneous reaction mixture often yields more consistent results and should be favored in fundamental studies.

The control of light irradiation intensity and reaction temperature are the primary responsible factors for the lack of reproducibility of photocatalytic procedures. In many setups, reaction heating originates from the light source, which prevents tight and constant control of the reaction temperature. Cooling the reaction with an external fan does not enable uniform temperature control, which is sometimes solved by using thermostats or cooling mantles. Poorly defined temperatures can have severe effects on the photocatalytic activity. For example, product selectivity may be lost due to competing reaction pathways. Further, temperature changes at the light source affect the homogeneity of irradiated light on long time scales; over the course of a long assay, the photocatalytic outcome may be affected. Some reactions only experience a photothermal effect, which makes proper temperature sensing and control highly important to distinguish between different effects of irradiation.^126^ Nonetheless a potential solution for insufficient heat transfer is presented by flow chemistry providing a high surface-to-volume ratio.^118^ With this technique in hand, chemical incompatibilities transform into an engineering challenge, thus necessitating interdisciplinary efforts. Similarly, heat transfer issues in high-throughput experimentation may benefit from (stopped-)flow chemistry where each droplet could render a new parameter set.^127−129^ Importantly, lack of control of light wavelength and intensity and temperature affect the reproducibility between different laboratories and sometimes even within the same group.^3^ When designing the photocatalytic methodologies, adding a detailed description of the irradiation set up and reaction parameters used is needed when reporting them to the field.

Furthermore, to ensure homogeneous and constant light irradiation wavelength and intensity, it is highly important to use refrigerated LEDs that keep a constant temperature. This is because the emission intensity of LEDs typically decreases with increasing temperature.^130^ Only a few commercial and custom-made photoreactors currently take this issue into account, even though it is especially critical when performing kinetic and other mechanistic studies. In such experiments, even small changes in the intensity and homogeneity of the light irradiation can lead to non-reproducible or misleading results.^3,118^

In this context, to facilitate the exchange of methodologies in the scientific community, it is of paramount importance to report measurable parameters that can help to normalize and standardize the photocatalytic procedures. Commercial and standardized equipment is available and recommended^117^ but sometimes does not meet the specific reaction requirements. The community will benefit from reporting the light intensity and the number of photons that have been used per reaction and the respective quantum yield of the underlying process.^114,115^

## Merging Photochemistry and Catalysis

Once individual mechanistic steps are well understood, one can combine photocatalytic cycles in one pot with other catalysts to increase the complexity of reaction schemes and products, while reducing the number of sequential transformations and intermediate isolations. Photocatalysis has been successfully combined with the three pillars of catalysis, namely, transition metal catalysis, organocatalysis, and biocatalysis. The use of catalysis in confined spaces draws inspiration from the supramolecular community and is also harnessed in the field of artificial photosynthesis to mimic complex biological systems. The interconnection of these disciplines outlines the interdisciplinarity necessary to develop new approaches.

### Metallaphotoredox Catalysis

Since the rediscovery of organic photocatalysis in 2009 by MacMillan,^131^ Yoon,^132^ and Stephenson,^133^ a vast number of catalytic transformations have been developed. MacMillan and Doyle’s groundbreaking work on the merger of nickel and photocatalysis^122^ opened the realm of metallaphotoredox catalysis. The field has greatly expanded the capacity of (abundant) transition metal catalysis, enabling elusive cross-coupling and transformation from readily available and native functional groups by harvesting light.^41^ The access to uneven redox states of the transition metal catalysts became available through SET processes and allowed the use of first-row transition metals such as iron, nickel, and copper.

From a synthetic perspective, the novelty of photoredox catalysis lies in its ability to activate nontraditional nucleophiles via a single electron transfer event, resulting in rapid access to radical intermediates. Subsequently, incorporation of the corresponding radical species into the transition metal catalytic cycles achieves transformations with reluctant electrophiles by modulating the metal’s oxidation state. Currently, the surge in investigating the abundance of metals, especially inexpensive first-row transition metals, nourishes the frontier of organometallic transformations.

**Scheme 2:**
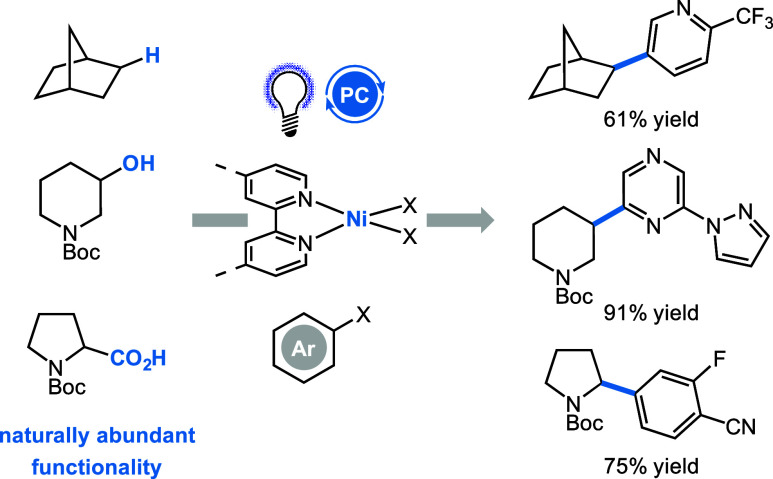
Arylation of Abundant Functional Groups with Nickel Photoredox Catalysis^122,134,135^

The widespread success of nickel catalysis, combined with photocatalytic activation, expands the toolbox of C(sp^2^)–C(sp^3^), C(sp^3^)–C(sp^3^), and C(sp^2^ or sp^3^)–heteroatom cross-coupling. Readily available carboxylic acids, halides, or even alcohol functional groups are used as the radical precursors (Scheme 2).^57,135−137^ Additionally, direct sensitization of higher valent nickel complexes facilitates reductive elimination steps and circumvents the use of additional photocatalysts in the reaction mechanism.^138,139^ Copper, which is less toxic than Ni, is an appealing alternative^140^ and also enables powerful C(sp^2^ or sp^3^)–heteroatom cross-coupling reactions.^141,142^ The unique character of the Cu-dual catalysis lies in its Lewis-acidic nature, which facilitates the reaction of alkynes with weak nucleophiles and enables “auxiliary ligand-less” cross-coupling.^143−145^ The mechanistic diversity of both nickel and copper photocatalysis is often not fully resolved and part of recent studies regards possible activation modes like (triplet) energy transfer or single-electron transfer.^146,147^ Besides, cobalt catalysis has attracted attention for producing (un)saturated compounds, which is a result of the high basicity of Co(I) or Co(II) favoring the hydrogen abstraction or transfer process.^148−150^ However, a limitation with respect to Cu- or Co-catalysis remains, namely, the additional substrate activation and the need for external stoichiometric reductant or oxidant.^149,151,152^ Another noteworthy aspect of these three metals is their capacity to initiate light-induced homolysis to generate radicals directly from starting materials.^153−156^ In particular, the homolysis of Cu(II)–Cl complexes has been extensively used to enhance the (di)functionalization of unsaturated systems such as alkenes, alkynes, and imines.^157^ Despite initial success in the fluoroalkylation of olefins^158^ and the deracemization of alcohols,^159^ future work should prioritize earlier transition metals, like iron or titanium, which are likewise challenging to engage in single-electron transfer reactions.^36,160^

In addition to increasing reactivity and promoting reactions that are not otherwise possible, an important feature of catalysis is the control over the outcome of a reaction, such as enantioselectivity (*vide infra*). Chiral ligands have proven to be successful in metal-catalyzed reactions but are not available for direct coupling procedures. For photocatalytic approaches, the use of chiral Lewis acid catalysts, organocatalysts, and biocatalysts has been successful for obtaining enantioenriched products.

### Organo-photocatalysis

**Scheme 3:**
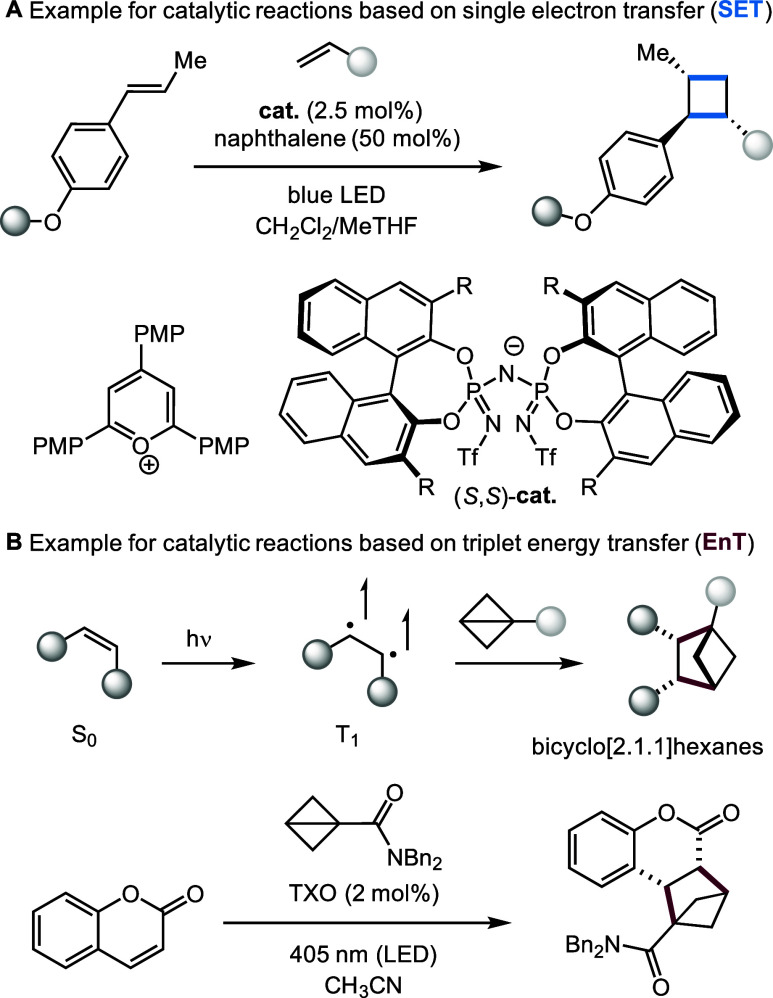
Selected Examples of Organocatalytic [2 + 2] Reactions Operating under SET to Obtain Cyclobutanes (A)^164^ or EnT Mechanisms Obtaining Bicyclo[2.1.1]hexanes (B)^165^

An inherent challenge in photocatalysis is to achieve site- and stereoselectivity. Contrary to classical enantioselective catalysis, one has to fundamentally understand and control the catalyst–substrate interactions in the ground and excited states. The latter are typically short-lived, high-energy intermediates, which adds to the challenge. Early reports in the field of stereoselective photocatalysis use hydrogen bonding interactions (Bach) and covalent enamine formation (Nicewicz and MacMillan) for achieving enantio-control.^131,161^ In general, there are two main strategies for enantioselective photocatalysis: (i) the adaptation of organocatalysts previously established for thermal reactivity and (ii) the development of chiral photocatalysts. In the first category, it is often necessary to modify the (organo)catalysts to obtain photostability. In this context, Melchiorre and co-workers have developed fluorinated organocatalysts that have been successfully applied in enantioselective photoreactions.^162,163^ The combination of ion-pairing with chiral organocatalysts was achieved by the List group (**cat**. in Scheme 3A). They showed that single-electron oxidation of the styrene derivative occurs with an achiral pyrylium photocatalyst. Enantioselectivity in the [2 + 2] photocycloaddition was controlled by the chiral anion.^164^

In the second category (designed chiral photocatalysts), Bach and co-workers have developed a large variety of reactions driven by a hydrogen-bonding catalyst with a covalently attached thioxanthone (TXO)-based triplet sensitizer.^51^

Beyond enantioselective transformations, they were also able to achieve deracemization reactions with chiral photocatalysts.^166^ It was also shown using a benzophenone photosensitizer, that enantiomeric synthesis of amino acids and dipeptides could be accomplished with this strategy.^167^ Current approaches in triplet energy transfer also include the use of bicyclobutane reagents, which Glorius reported for the preparation of bicyclo[2.1.1]hexanes (Scheme 3B).^165^ Hence the careful design of synthetic chiral organocatalysts or photosensitizers enables stereochemical control with high levels of selectivity. We note that chiral inorganic photocatalysts have been developed but are beyond the scope of this Perspective.^52^

### Photobiocatalysis

Enzymes offer a chiral environment with various types of tunable, noncovalent interactions to facilitate substrate–catalyst preorganization and the stabilization of reactive intermediates, which often leads to high stereoselectivity. In recent years, the field of photobiocatalysis has developed tremendously.^54^ Research has focused on understanding the mechanisms at play in naturally occurring photoenzymes, such as DNA photolyase^168^ or fatty acid photodecarboxylase,^169^ as well as engineering new photobiocatalyst systems. Additionally, powerful redox enzymes, such as cytochrome P450s, can be utilized in biophotocatalytic schemes by the transfer of photoinduced electrons.^170,171^ This strategy allows catalysis in the absence of natural redox partner proteins and their respective cofactors, a strategy that also inspires the field of artificial photosynthesis (*vide infra*). In addition, enzyme catalysis can also be combined with chemical photocatalysis in reaction cascades, such as the syntheses of enantiopure γ-substituted alcohols and amines from racemic β-substituted ketones.^54,172^

**Scheme 4:**
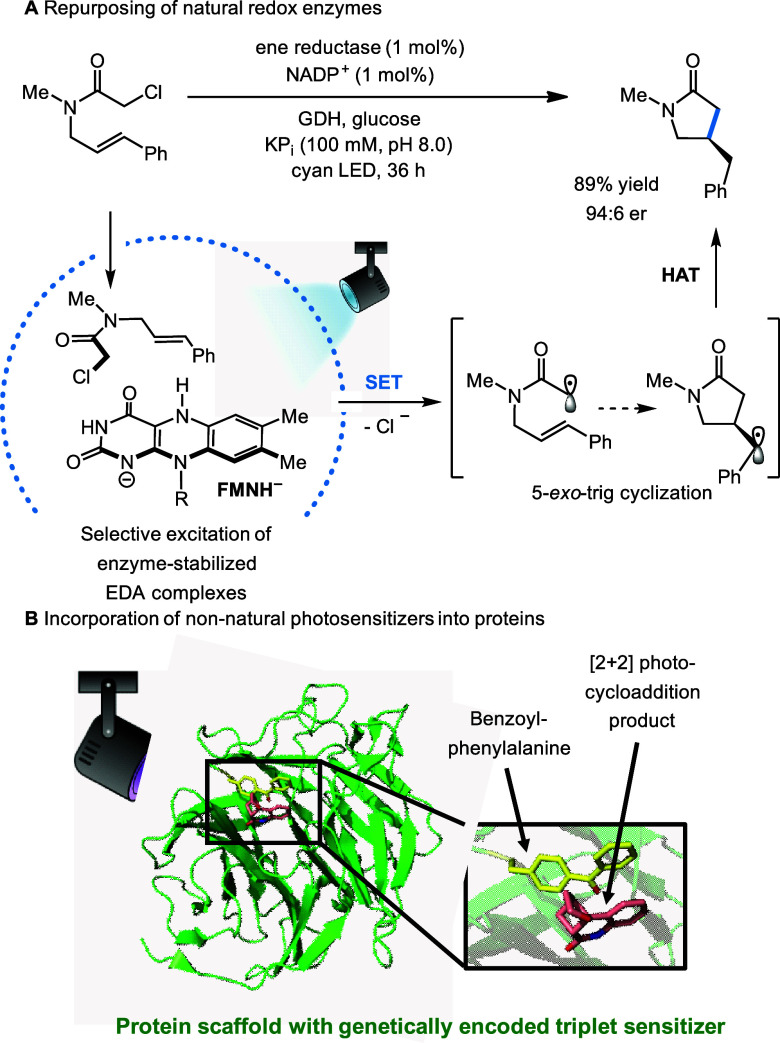
Selected Examples of Engineered Photoenzymes Based on SET from a Protein-Stabilized EDA Complex (A)^173^ and EnT from a Genetically Encoded Triplet Sensitizer Leading to 99% ee of the Product (B), PDB Entry 7ZP7(174)

The major challenge and opportunity in the field of biocatalysis is the development of artificial enzymes for reactions beyond Nature’s synthetic repertoire. It was recently demonstrated that natural redox enzymes can catalyze new-to-nature radical transformations with tight stereocontrol upon blue-light irradiation (Scheme 4A).^173,175^ Here, the protein can stabilize electron-donor–acceptor (EDA) complexes between the redox cofactor and a non-natural substrate. In a different approach, photoenzymes have been designed rationally by incorporating synthetic photosensitizers into proteins by either chemical modifications or genetic code expansion.^176−178^ For example, the noncanonical amino acid benzoyl-phenylalanine can be used as a genetically encoded triplet sensitizer, thereby generating artificial photoenzymes for stereoselective [2 + 2] cycloadditions (Scheme 4B).^174,179^ Furthermore, a computationally designed protein with a high-affinity lanthanide binding site was recently engineered to promote cerium-based photoredox catalysis.^180^

In all of these strategies, a key advantage of protein-based catalysts comes into play, namely, their evolvability. An initially low activity or selectivity can be significantly improved by directed evolution, which mimics natural selection in the laboratory.^181,182^ This concept has been extended successfully to light-driven enzymatic reactions. However, to fully exploit the potential of laboratory evolution, *in vivo* selection rather than *in vitro* screening of photoenzymes should be implemented in the future. This requires innovative strategies to couple the survival of a host organism to photoenzymatic activity. Considering the recent advances in de novo protein design and enzyme engineering, it is expected that photobiocatalysis will continue to gain importance.

### Photocatalysis in Confined Spaces

**Scheme 5:**
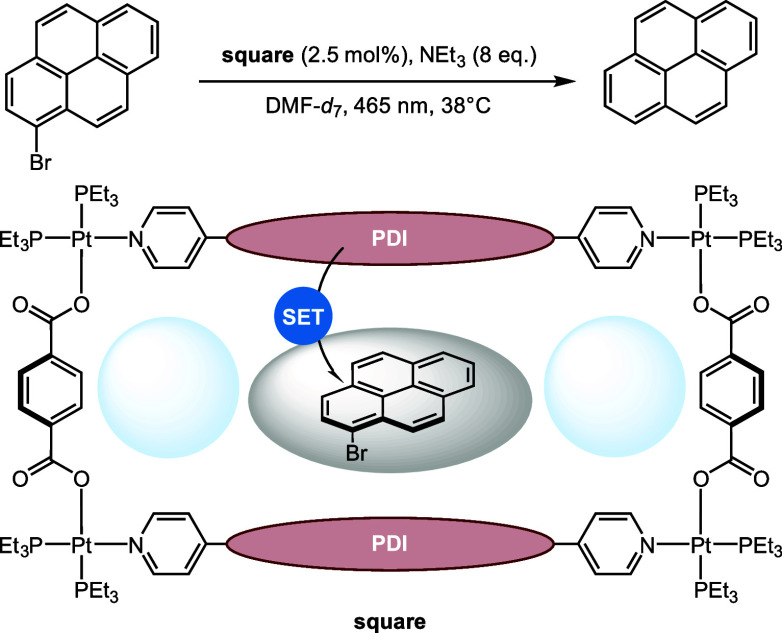
Supramolecular Square Containing Perylene-Diimide (PDI) Photocatalysts (red) That Can Be Used for Dehalogenation Reactions^187^ Substrates can be bound between the PDIs (gray), and the two outer pockets (light blue) could potentially accommodate radical scavengers to react with the generated radical intermediates.

For efficient and directional transfer of energy or single electrons, substrates and photocatalysts must be precisely organized, as the distance and orientation govern the reactivity. Such preorganization takes place in enzymes but can also be achieved in synthetic analogues such as micelles or supramolecular coordination cages.^20^ Coordination cages are synthetically accessible and modular and can be evolved as enzymes in a directed fashion due to the extension beyond the canonical amino acids.^183^ Synthetic supramolecular hosts provide a single cavity for binding of small molecules, which can be substrates as well as photocatalysts. Such systems have already been successfully applied for both artificial photosynthesis and organic photoredox catalysis.^20^ The use of supramolecular entities as reaction “containers” presents a potential solution to the challenges associated with radical coupling reactions. For example, early work by Nicholas Turro and co-workers demonstrates that two radicals that are similar in nature can couple selectively by using an aqueous solution of micelles as the reaction medium whereas as a statistical mixture is obtained in an organic solvent.^184^ Radicals generated through photoredox catalysis are typically short-lived and feature undirected reactivity. Supramolecular chemistry offers an attractive approach to address the challenge of undirected reactivity of radical intermediates by designing systems where radical intermediates and reaction partners are preorganized. This is achieved by dynamic covalent or noncovalent interactions to yield an additional level of control of the catalytic reaction.^20,185^ Common photocatalysts can potentially be integrated into supramolecular structures via metal-mediated self-assembly.^186^ For example, the Pullen group has recently demonstrated that when a PDI photosensitizer is incorporated into a supramolecular heteroleptic square, it maintains the ability to generate radicals from aryl halides (Scheme 5).^187^ Such generated radicals are highly reactive and form the dehalogenated products via hydrogen atom abstraction from solvent or sacrificial electron donors (e.g., NEt_3_). The supramolecular structure offers the potential to preorganize the halogenated substrate in proximity to the catalyst through binding via noncovalent interactions, facilitating efficient electron transfer and overcoming diffusion limitations.^188^ Furthermore, binding radical scavengers together with the halogenated substrates in the cavity would allow the use of highly reactive radical intermediates in C–C coupling reactions.

The redox potentials of a PC can be fine-tuned by supramolecular interactions between host and guest, which can for instance lower the overpotentials for the desired half-reaction, as has been observed for proton reduction catalysts encapsulated in a cationic coordination cage.^189^ Encapsulation or incorporation has also been shown to stabilize the catalytic intermediates, which allows for a more efficient process as the time difference of photophysical processes and catalysis can be bridged more easily.^190,191^

For the overall function of the assembly within a device, directional electron transfer becomes crucial. Supramolecular cages with encapsulated catalysts that allow spatial organization of the different components and thus directional electron transfer are therefore promising, especially if such a light-absorbing cage can be immobilized on an electrode surface. The latter is rather unexplored in the context of photocatalysis, though immobilization strategies for coordination cages on surfaces via electrostatic^192,193^ and hydrophobic interactions exist.^194^

In organic photocatalysis, supramolecular cages can contribute to substrate and product selectivity, as well as to enhance the catalytic rate in a similar fashion as enzymes, since they are able to discriminate different guest molecules and can increase the local concentration of substrates around the active site.^195^ Furthermore, encapsulation of substrates induces spatial constraints, which affect the productivity of the reaction. Moreover, a chiral cavity is able to transfer the chirality to the reaction products, which allows for enantioselective photoredox catalysis.^196^ However, it is key to understand the ground state host–guest equilibria and their interactions to achieve the maximum impact from the supramolecular strategy.

Immobilization of supramolecular cages containing photocatalysts on electrode surfaces will allow one to drive electron-primed photoredox catalysis (*vide supra*), in combination with utilizing second coordination sphere effects of the supramolecular cage to preorganize substrates and direct reactivity of short-lived intermediates more specifically.^197^ Furthermore, one could imagine that the immobilization of supramolecular cages will allow their integration into flow reactors. The main limitations currently are the formation of stable coordination cages that can be rigidly linked to electrodes without leaching or degrading under applied potential during electrocatalysis.

### Artificial Photosynthesis

Natural photosynthesis and other catalytic processes in nature have evolved to catalyze a wide range of chemical reactions with high fidelity and efficiency and low overpotentials; their efficacy lies in their superb ability to organize the delivery of substrate and redox equivalents to the catalytic site spatially and temporally. In addition, it has been increasingly recognized that natural systems utilize proton-coupled electron transfer (PCET) to lower activation energies for redox processes.^198,199^ Nature’s catalysts, therefore, serve as an important blueprint in the development of artificial photocatalytic systems but also for synthetic analogues of enzymes that may perform other reactions.

Artificial photosynthetic systems aim at utilizing solar energy as the sole source of energy to transform thermodynamically stable and plentiful reactants such as H_2_O, N_2_, and CO_2_ into energetic fuels and feedstocks such as hydrogen, ammonia, methane, ethane, and other carbon-based chemicals. Artificial photosynthesis is a direct, or potentially “wire free”, method that provides a pathway to a sustainable and circular carbon economy that has the potential to play a major role in mitigating the widespread use of fossil fuels.^200^ While artificial photosynthesis primarily focuses on the conversion of small molecules, the elementary steps (i.e., SET and PCET) are akin to those of organic photoredox catalysis.

**Figure 4:**
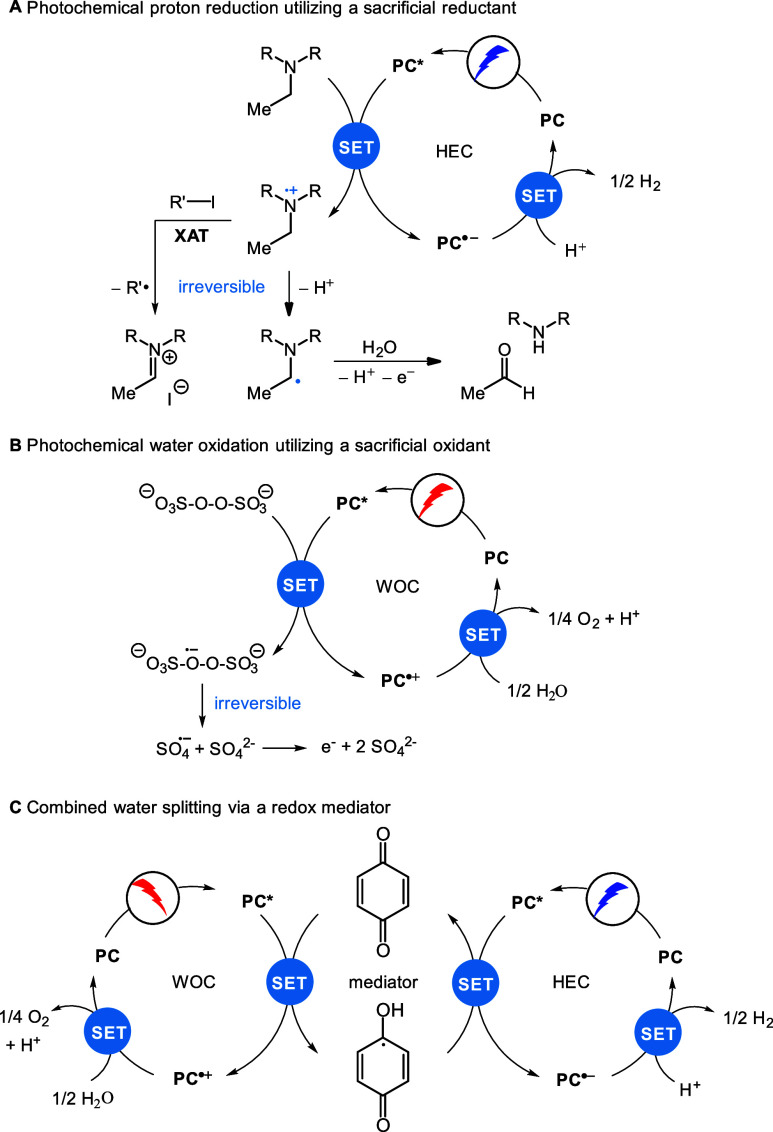
(A) Photocatalytic proton reduction is often driven by the irreversible N–C bond cleavage in the tertiary amine sacrificial electron donor (here, triethylamine). (B) Photocatalytic water oxidation (WOC) with persulfate as a sacrificial oxidant. (C) Quinone redox mediators may be used to couple both reactions and thereby prevent the use of sacrificial reagents.

An overwhelming number of publications report on half-redox reactions: either on the oxidative side, where the photosensitizer and catalyst aim at oxidizing a substrate such as water, hydroxide ions, or organic substrates, or on the reduction side, where the target reaction is the photoreduction of CO_2_, protons, N_2_, or organic substrates. In both cases, a sacrificial reagents must be used to provide redox equivalents to drive photocatalysis:^201^ either electron-accepting agents, such as [Co(NH_3_)_5_Cl]^2+^, periodate, or peroxodisulfate for the photo-oxidation side or electron-donating agents such as amines, ascorbate, phosphines (e.g., tris(2-carboxyethyl)-phosphine, TCEP), or thiols on the photoreduction side. On the one hand, studying half-reactions has allowed the community to simplify the problem of artificial photosynthesis and advance photocatalysis research significantly. For example, constant progress in the catalytic activity of water oxidation, water reduction, or CO_2_ reduction catalysts occurred using photocatalytic half-reaction systems. Before artificial photosynthetic strategies are realized and scaled up, these optimized catalysts need to be coupled to excellent photosensitizers that are stable and capture a large proportion of the solar spectrum. On the other hand, in most photocatalytic systems developed for solar fuel generation an irreversible bond cleavage in the photoreduced electron acceptor or photooxidized electron donor is actively limiting charge recombination and, in fact, driving the photocatalytic reaction toward O_2_, H_2_, or carbon fuel generation. For example, the central O–O bond in peroxodisulfate breaks irreversibly upon accepting an electron,^202^ while tertiary amines losing an electron form radical cations that end up irreversibly cleaving a C–N bond to afford an aldehyde and a secondary amine (Figure 4).^203^ In both cases, the irreversibility of one of the elementary steps of the photo-oxidized sacrificial electron donor or photoreduced sacrificial acceptor drives the unidirectionality of the photocatalytic half-reaction. The reactivity and irreversibility of amine cleavage have been productively applied in halogen atom transfer (XAT) catalysis. Leonori et al. have developed impressive applications in organic transformations and cross-coupling using amines.^43,204^ It should be noted that stable tertiary amines, with C–N bonds remaining intact, found widespread applications as sacrificial electron donors in organic transformations ranging from hydrogen atom transfer (HAT) to radical aromatic substitution (radical S_N_Ar) reactivity to name only a few.^205−207^ More research into reaction systems in which both half-reactions are coupled is desirable, and although challenging, it will likely result in novel types of chemical reactivity and applications in the context of both artificial photosynthesis and synthetic photoredox catalysis.^208−210^

Of course, it is impossible to drive the world’s solar fuel demand with the massive production of peroxodisulfate, amines, or ascorbate, and from a sustainability point of view, the use of sacrificial reagents is wasteful and must be avoided. For artificial photosynthesis to contribute to green processes, we must use a more widely available source of electrons, such as water, and couple photooxidation and photoreduction half-reactions electronically with respect to protons. Most existing artificial photosynthetic systems capable of coupling water oxidation to the reduction of protons or CO_2_ use a conducting wire,^211−215^ while the transfer of protons is mediated through the solution or a proton-conductive membrane. In principle, in artificial photosynthesis, the coupling between the oxidation and reduction half-reactions could also use reversible electron relays, or redox mediators, to transfer the photogenerated electrons and protons. There is a surprising lack of understanding of how molecular electron relays can be used to achieve the same effect.^216^ This knowledge gap is unexpected given that in natural photosynthesis, which has been studied for decades, electrons are brought from one side of the thylakoid membrane to the other precisely via molecular redox mediators present in both oxidized and reduced forms in the mixture: either quinones/hydroquinones, iron–sulfur clusters (ferredoxin), and/or NADP^+^/NADPH.^217^ Though mixtures of oxidized and reduced electron relays such as iodide/iodine or cobalt complexes have been used to close the redox cycle in dye-sensitized solar cells,^218^ and the (photo)chemistry of quinones is well-documented,^219^ during recent years, a number of artificial photosynthetic studies have been performed using such mixtures of electron relays to couple two half-redox reactions.^220^ We are persuaded that this lack of effort hinders the understanding of charge recombination kinetics in full artificial photosynthetic systems, thereby limiting the development of practical devices toward efficient solar fuels production.

Bioinspired catalytic systems can vary widely in their physical form, from systems that do not resemble Nature in a physical sense yet mimic catalytic behavior (e.g., photosynthetic MOFs or discrete supramolecular cages)^221^ to existing natural systems that have been re-engineered to enhance inherent catalytic activity^222^ or produce non-natural chemical products.^223^

Between these two extremes lie hybrid systems, where catalysts are designed to combine natural and artificial components. Several intriguing hybrid systems feature a catalytic center bound into a natural or designed protein scaffold, where the protein can (i) impart water solubility to the catalyst, (ii) protect the catalyst from unwanted side chemistry, and (iii) be readily modified to functionally support H^+^ and e^–^ flux of catalysis.^224−227^ Such hybrid systems are typically smaller than natural enzymes, meaning that their greater atom economy increases their feasibility for scale-up. Regardless of the approach to artificial photosynthesis, research in the field will need to address how to improve catalyst performance to optimize electron transfer and PCET, have long-term stability, and use only earth abundant elements. Before artificial photosynthetic strategies are widely realized, optimized catalysts need to be coupled to excellent photosensitizers that are stable and capture a broad wavelength span, which allows capturing a large proportion of solar energy. Several interesting examples of functioning coupled artificial photosynthetic systems that were powered solely by sunlight have been developed to date.^221,228^

To sustain the rapid development of photochemical processes in a variety of settings and systems and potentially formulate design principles for reaction development, a deeper understanding of the underlying mechanisms is important, as outlined above. Additionally, in combination with rather low energy sunlight, the efficiency of artificial photocatalytic systems decreases. A fine balance between energy consumption for artificial light sources and solar radiation for improved sustainability must be evaluated.

### Microfluidic Photocatalysis

Reactor design is especially important in photocatalysis. Traditional batch reactors often suffer from inefficient and inhomogeneous light penetration, limiting the potential of photocatalysis simply because catalysis is then limited by the availability of photons that are needed to activate catalysis. Therefore, microfluidic reactors have attracted enormous attention, as they allow for better light penetration due to significantly smaller reactor diameter.^118,229^ Capillary reactors, for instance, achieve significantly better yields in shorter reactions times than batch reactors due to more homogeneous irradiation. For example, de Oliveira and McQuade demonstrated that a capillary reactor performed the photooxidation of naphthol derivatives at significantly higher yields (up to 82%) in comparison to a batch reactor (up to 20% yield), while simultaneously accelerating the reaction time to 5 min from 120 min for the batch system.^230,231^ They utilized a tetraphenyl porphyrin photosensitizer (TPP) to generate singlet oxygen for this reaction. This example also demonstrates the safe handling of hazardous or challenging compounds such as gases, which is more difficult in traditional batch reactors.

In view of the integration of automation and machine learning in organic synthesis, microfluidics may provide an additional advantage: high-throughput experimentation platforms based on flow reactors allow the rapid handling and evaluation of multiple reactions in parallel, thus accelerating the reaction discovery. Crucial for this is the development of accurate and fast in-line analytics, such as IR sensors, NMR, and Raman.^232^ In the future, fully automated robotic platforms such as the recently developed RoboChem by the Noël group may execute synthesis screening and optimization.^129^

## Photocatalysis as a Green Technology?

While water is considered a green reaction medium for chemical processes,^233^ conventional chemical processes often rely on toxic or volatile organic solvents that pose significant risks to both human health and the natural environment.^234^ Despite this, if water becomes tainted with toxic substances, the cost of wastewater treatment is often so high that the entire process may not be economically viable. Additionally, purifying an aqueous solution from organic contamination might require more energy compared with purifying organic solvents.

Given the limited water solubility of highly active yet hydrophobic photocatalysts, such as traditional iridium-based (e.g., *fac*-[Ir(ppy)_3_]) and organic photocatalysts (e.g., phenothiazine and perylene diimide), various strategies have been developed to enable light-driven reactions in aqueous settings. Apart from making photocatalysts water-soluble through chemical modification,^235,236^ successful approaches include bimolecular π–π-stacking of reagents combined with hydrogen-bonding^237^ or their encapsulation within micellar systems,^238^ nanosized molecular capsules,^239^ and polymeric nanoparticles.^240−242^

In addition to its environmentally friendly nature, water possesses distinct characteristics that render it a highly valuable solvent by influencing chemoselectivity,^243^ boosting photocatalyst activity,^244^ and contributing to the lowering of energy levels in chemical processes.^245^ Incorporation of water as a green solvent hence seems feasible, and photocatalytic methods have the potential to align with the general principles of green chemistry.

**Scheme 6:**
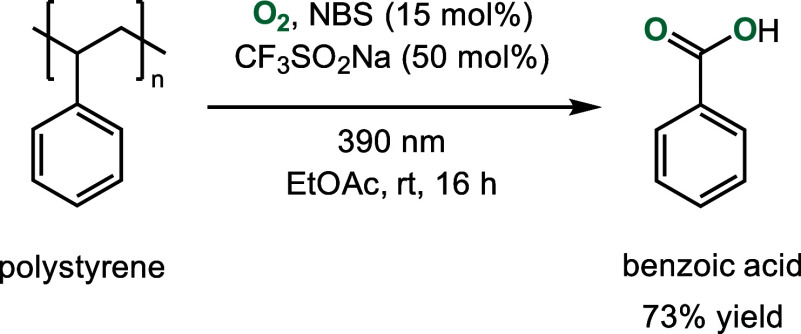
Photocatalytic Upcycling of Commercial Polystyrene into Benzoic Acid under Metal-Free and Scalable Conditions Facilitated by Orchestrated HAT Events^18^

In recent years, there has been a surge of interest in the photocatalytic deconstruction of plastics due to researchers recognizing its immense potential in effectively addressing the global crisis of plastic pollution.^58,59^ With plastic waste littering landfills and polluting oceans, photocatalysis emerges as a promising solution by breaking down polymers into their molecular components through selective scission of robust chemical bonds within the polymer backbone. Various reported protocols target a wide range of synthetic macromolecules, including hydroxylated polymers^17,246,247^ and polystyrenes (Scheme 6).^18,19,248,249^

Furthermore, photocatalytic valorization of biological polymers, such as lignin^250,251^ and cellulose,^252^ has become an aspiring approach in this field. In both cases, valuable building blocks are retrieved, which can be seamlessly integrated into existing chemical processes, thereby reducing the demand for virgin petrochemical resources. As a result, photocatalysis offers a sustainable approach toward repurposing plastic and biological waste materials, contributing to waste reduction and resource conservation efforts.

Nitrogen-based fertilizers are generally obtained through hydrogen production, followed by dinitrogen activation at high pressure in the Haber–Bosch process. Replacing this energy intensive process with a milder photochemical process, ideally under ambient conditions, would be an industrial milestone. However, recent advancements in the area of photochemical nitrogen activation are still facing significant challenges in catalyst choice and mechanisms.^253^ In particular late transition metals and high energy UV light are utilized as exemplified by Schneider et al. for an efficient nitrogen-to-chemicals process.^254^

**Figure 5:**
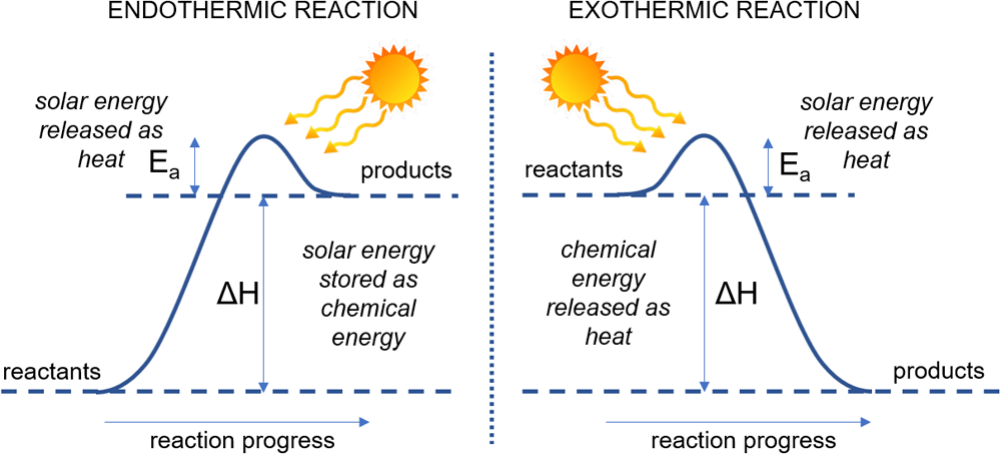
Simplified energy diagram for chemical reactions driven by solar energy, a key consideration in the energy transition.

Nowadays, the chemical industry depends mainly on the use of fossil fuels for both the energy and feedstock supply. The burning of these hydrocarbons to generate heat for chemical reactions results in substantial fossil CO_2_ emissions. As outlined above, photochemistry lately has received more attention due to concerns regarding irreversible climate change, partly caused by increasing atmospheric CO_2_ levels. Photochemistry is seen as an approach to increase the use of renewable energy to produce fuels and chemicals and, thus, to avoid the use of fossil energy and related CO_2_ emissions. Indeed, photochemistry can play a role in novel methods that can convert or even store energy in molecules.^255^ Here it is important to note that in order to store energy it is necessary to drive endothermic reactions by light (Figure 5). Only then will part of the solar energy be stored (Δ*H* > 0) in the newly formed molecular bonds. In exothermic reactions, chemical energy is released (Δ*H* < 0), and at best, only the activation energy (*E*_a_) can be provided by solar energy. In reality, this latter process often requires active cooling, and adding more heat by means of light seems unnecessary and even inefficient. In particular decarboxylative transformations are powerful but generate stoichiometric amounts of CO_2_.^45,137^ In some cases even preactivation is required, adding an economic burden onto the reaction equation, which needs to be considered from an environmental point of view.

In the end, the commercial feasibility of photochemical processes will largely depend on their competitive advantage over alternative approaches. Solar fuel production competes with other renewable energy technologies and, as such, should be scalable and cheap.^21^

The production of fine chemicals results in molecules with a higher value, but the potential impact on climate change and renewable energy supply is significantly smaller. For this category, energy efficiency and unique chemical reactivity seem to be the best possible selling points.

The power density of solar light is relatively low. This means that a substantial geometric surface area is required to harvest light and use it for photochemistry. A large surface area means that considerable amounts of materials are necessary, typically leading to higher investment costs. The large surface area also offers an advantage in that photochemical devices will likely be engineered as modules. These modular devices can be produced through mass manufacturing processes and allow a scale-up by numbers. These characteristics may result in a steep learning curve and fast cost reductions,^256^ as is also observed for solar photovoltaics (PVs).^257^ The use of artificial light sources or solar generators may enhance the power density, which would reduce the required surface area of the photochemical device. Such a process seems attractive but suffers from severe energy losses on going from renewable energy to electricity to artificial lighting. Early stage techno-economic assessments can help to identify the most attractive photochemical processes and steer innovation and developments in the right direction.

## The Future of Photocatalysis

In a future where renewable energy supplies and circularity will reign, the production of chemicals and fuels is also likely to become more decentralized. Such “chemical and energy centers” are considered to be smaller in capacity than conventional chemical plants so as to more efficiently connect better to feedstocks and energy supplies. This leads to the following questions: How can biomass and societal waste be converted into valuable feedstocks or products? How can renewable energy be optimally used to drive chemical processes? To answer these questions, photochemistry is one avenue to investigate in more detail. This will require substantial developments in the field, both to demonstrate the capabilities of photochemistry in an industrially relevant environment and to achieve a deeper fundamental understanding of the different photochemical processes and their opportunities.

Photocatalysis can play an important role in the development of sustainable methods. Inspiration from natural processes is and will be a key guidance toward this goal.^258^ The field has the potential to use sunlight for making and breaking molecular bonds for synthesis and deconstruction of molecules and materials as already envisioned more than 100 years ago.^9^ The use of abundant metal catalysts, enzymes, artificial photosynthetic systems, mechanistic studies, and photoreactor homogeneity could all assist the development of future-proof processes and enlighten the role of photochemistry for sustainability. However, photocatalysis is still often treated as a black box, since mechanistic variability and limited knowledge of mechanisms reduce the ability to rationally develop novel synthetic routes. Furthermore, a deep mechanistic understanding combined with spectroscopy-guided optimization can lead to improved reaction quantum yields, which will accelerate the transition from laboratory-scale to industrial applications.

Photoredox strategies have facilitated increased access to radical formation, especially stabilized radicals. Additionally, most reported examples rely on additional driving forces provided by, for example, gas extrusion or high molecular weight leaving groups as redox handles. While these methods present valuable advances in the field, it remains challenging to avoid substrate engineering, couple transient radicals, or use less-stable radicals in productive chemical transformations. Within synthetic photocatalytic strategies, controlling the enantioselectivity is an inherent key challenge due to the involvement of high energy intermediates. Here, biocatalysis has proven to be a promising strategy to synthesize highly enantioenriched complex molecules. Further developments allow for novel reactivity that leads to stereo- and regioselective reactions beyond the current state of the art and closer to enzymatic performance. Combining biologically relevant enzymatic catalysis with visible light redox catalysis enables new-to-nature reactivity, which is accessed from excited states and impossible by traditional ground-state reactivity. A key challenge is the implementation of *in vivo* selection systems for photoenzyme evolution in the lab. If the survival of a bacterial host could be coupled to a photoenzymatic activity of interest, directed evolution is more efficient than screening single mutants in 96-well plates.

Although photocatalysis inherently provides high energy intermediates, there is an interest in increasing reactivity. Reaching the highest amount of energy with the use of the lowest energy light sources is a persistent challenge in photochemical method development. Alternatively, electrochemically mediated photoredox catalysis, which first generates radical anions or cations followed by excitation, leads to excited states with extreme redox potentials and can facilitate new reactivity.^112,259^ Another approach utilizes triplet–triplet annihilation (TTA) upconversion as a strategy to combine the energy of two photons to obtain a higher energy species. These high energy intermediates will allow otherwise challenging ground-state reactions if new photocatalysts, which are susceptible for photoelectrochemical activation or TTA upconversion, are developed. Mechanistic understanding and development of upconversion^260^ or multiphoton absorption^87^ processes are important for further photocatalyst design. In combination with organic synthesis, novel (cascade) reactivity will become accessible through the use of low-energy photons. Hence, understanding the photochemical mechanisms and photophysical properties is of the utmost importance.

Lastly, differences in photoreactor systems and reproducibility of experiments have arisen as a timely challenge for a productive and sustained development. Reactor design and proper detailed reporting protocols are often not met, which limits reproducibility and reliability.^261^ In this context, a standardized guideline on reporting data across laboratories and across disciplines must be outlined.^262^ Since the field of photochemistry has grown so quickly over the last two decades, updated guidelines from 1982 and 2006 are necessary.^263−265^

In the future, multiple relatively small flows of materials, either based on biomass or municipal/industrial waste, that have to be reused are a likely scenario. This may require flexible, sustainable routes and technologies that can convert these feedstocks, ideally in proximity to where the feedstock is generated to limit energy losses due to transportation. Additionally, this will also allow for easier coupling of a renewable energy supply to conversion processes as it is generated more locally compared to, for instance, crude oil, which is transported around the world in bulk. Such processes are likely to benefit from new types of (photo)reactivity and (artificial) light-driven chemistry engineered in new types of (microfluidic) flow reactors which can be manufactured highly modularly and in mass. An important factor to consider in the context of biomass or waste conversion is the fact that these are impure feedstocks to begin with. If one wants to avoid exhaustive prepurification steps, the photocatalysts used to convert these feedstocks selectively into specific products will have to be highly substrate specific, as multiple potential substrates will be present in the same reaction mixtures. Some of the components present in the mixture may deactivate or degrade the catalysts. Others may lead to unwanted side products, limiting the overall yield of the target products. This is especially relevant for photocatalysis involving radical intermediates that are highly reactive and may, for instance, abstract a hydrogen atom from solvent or other components in solution instead of undergoing a C–C coupling reaction or similar. Supramolecular chemistry and enzymes will play a predominant role in this context, as they offer the possibility to preorganize specific substrates and reaction partners in proximity to the photocatalyst, thereby enabling selective light-induced energy or electron transfer.

A main challenge in artificial photosynthesis is to rapidly liberate electrons needed for proton- or CO_2_-reduction, together with the formation of C–C bond containing products derived therefrom. In natural photosynthesis, electrons are generated via water oxidation, a reaction that creates one of the main bottlenecks in artificial photosynthesis. Development of inexpensive, stable, and easy-to-make photocatalysts facilitates this goal.

## Concluding Remarks

Overall, the future of photocatalysis is promising, especially when disciplines are merged to enhance sustainable chemical processes. The focus lies in improving the mechanistic understanding and reaction quantum yields to extend photocatalysis to industrial applications. Future research should deepen our understanding of photocatalytic systems, optimizing performance and reproducibility to design more efficient systems, potentially revolutionizing energy storage, waste treatment, and synthetic chemistry.

Lastly, the development of novel fundamental concepts collides with the rapid developments urged by the energy transition. Room for interdisciplinary photochemical research must be retained to enable out-of-the-box thinking to meet our many economic and ecological demands. Emphasizing fundamental research is crucial to ensure that innovation continues to flourish, allowing for comprehensive solutions that address both immediate applications and broader scientific challenges. Where is the room for fundamental research ideas if we only rush toward applications?
